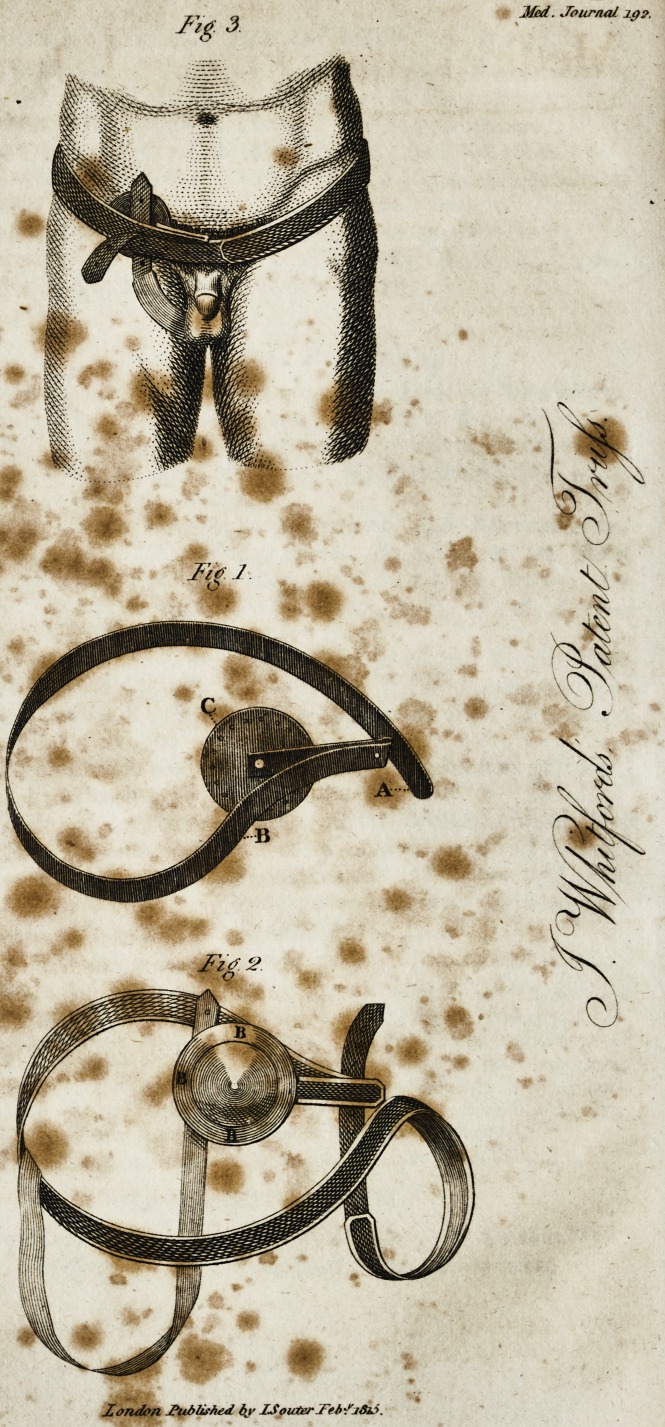# Remarks on the Case of W. O., with Suggestions for Its Cure

**Published:** 1815-02

**Authors:** Leyson Rees

**Affiliations:** Member of the College of Surgeons in London. Merthyr Tydfil, Glamorgan


### Medical and Physical Journal.

2 OF VOL. XXXIII.] FEBRUARY, 1815. [NO. 192.

"For many fortunate discoveries in medicine, and for the detection of numerous errors, the world is indebted to the rapid circulation of Monthly Journals; and there never existed any work to which the faculty in Europe and America were under deeper obligations than to the Medical and Physical Journal of London, now forming a long, but an invaluable series."—Rush.

*For the Medical and Physical Journal.*

### *Remarks on the Case of W. O., with Suggestions for its Cure;* by Mr. Leyson Rees.

I SHOULD not have ventured at this time to have offered my opinion on a mode of cure for a disease, which has perplexed our first medical practitioners, if I had not been induced to do so by a letter in your Journal for this month from your correspondent W. O.

I will not pretend to say that he has never had the venereal disease, but, from his own account, I have no hesitation in believing that he has not now. It has occurred to me, in several instances, to see many cases of this disorder; and I can venture to say they have, in every case, yielded to one treatment; and, if it were not inconvenient to describe cases where the sufferers would be known from the account, I could mention several very similar to your unhappy correspondent, labouring for instance under ulcerated nodes on the tibia and cranium, blotches on the skin, ulcerated throats, with loss of the velum pendulum palati, and even of the bones of the palate, &c. nocturnal pains; in short, every symptom of the venereal disease, excepting, in some of the cases, the absolute impossibility of its ever having been communicated. All these symptoms I have known yield to the remedy I use, without a particle of mercury, or any other usually antisyphilitic remedy being taken. I think no other proof of these diseases never having been venereal need be offered.

My plan of treatment consists, after first paying attention to the state of the stomach and bowels, in administering steel in as large a proportion as can be given. The preparation I generally use is the sulphat of iron, in the form of the compound mixture of iron, or the pills of iron with myrrh myrrh of the last London Pharmacopœia; but I believe any other preparation of steel equally efficacious.

It may be objected, that the state of the pulse will not, in all cases, admit of this remedy. I can only say I have not found it so; and, as the disease, in every case I have seen, is attended with great debility and emaciation, it is not likely to be found so. I have recommended a trial of the medicine to a medical friend, who, in two instances, has experienced the most beneficial effects to result from its use.

I have not mentioned auxiliaries, such as opium, &c. &c. of course every practitioner will administer them as occasion may require.

As Phlegmasia Dolens appears to excite some notice at present, I beg to observe, that I have just had a case, in a young married woman, *her first child*, which yielded to warm and anodyne fomentation to the whole limb, and very gentle frictions upwards; but, as the patient was suffering considerably under the effects of a severe hæmorrhage consequent on adhesion of the placenta, the antiphlogistic regimen was not adhered to,—indeed her pulse could not have admitted it. The disease made its appearance six or seven days after delivery. There was little or no milk secreted. The child died half an hour after its birth.

LEYSON REES,

*Member of the College of Surgeons in London.*

*Merthyr Tydfil, Glamorgan,*

*Dec.* 16, 1814.

---

*For the Medical and Physical Journal.*

## Figures and Tables

**Fig. 3. Fig. 1. Fig. 2. f1:**